# How to tell time: advances in decoding circadian phase from omics snapshots

**DOI:** 10.12688/f1000research.26759.1

**Published:** 2020-09-17

**Authors:** Lorenzo Talamanca, Felix Naef

**Affiliations:** 1 The Institute of Bioengineering (IBI), School of Life Science, Ecole Polytechnique Fédérale de Lausanne (EPFL), Lausanne, Switzerland

**Keywords:** circadian rhythms, chronomedicine, omics, data mining, chronobiology

## Abstract

The ability of organisms to keep track of external time, by means of the circadian clock interacting with the environment, is essential for health. The focus of this review is recent methods to detect the internal circadian time of an omics sample. Before reaching our main topic, we introduce the circadian clock, its hierarchical structure, and its main functions; we will also explain the notion of internal time, or circadian phase, and how it differs from the geophysical time. We then focus on the role played by the clock in the maintenance of human heath, in particular in the context of cancer. Thereafter, we analyze an important methodological question: how to infer the circadian phase of unlabeled omics snapshot measurements. Answering this question could both significantly increase our understanding of the circadian clock and allow the use of this knowledge in biomedical applications. We review existing methods, focusing on the more recent ones, following a historical trajectory. We explain the basic concepts underlying the methods, as well as some crucial technical aspects of each. We conclude by reporting how some of these methods have, more or less effectively, enabled furthering our understanding of the clock and given insights regarding potential biomedical applications.

## Introduction

Earth rotation around its axis imposes 24 hour rhythms to all life on the planet. The circadian, from Latin “circa diem” (about a day), clock ^1^ is the celebrated evolutionary response to this intrinsic periodicity of the environment. The circadian clock is a molecular and pervasive ^2^ cell autonomous oscillator. This clock gains its periodicity of almost 24 hours ^3^ from an as-yet incompletely understood mechanism, which most likely involves a transcriptional–translational negative feedback loop ^4^ but probably also other regulatory layers. In mammals, the structure for time-keeping is hierarchical ^5^: a master clock ^6^ is hosted in the brain’s suprachiasmatic nuclei (SCN) and peripheral clocks tick in virtually all of the other organs. The clock in the SCN responds to the external light cycle and synchronizes the body’s peripheral clocks ^7^. However, these peripheral clocks are susceptible to temporal cues different from light ^8^, e.g. the clock in the liver is responsive to feeding patterns ^9^. The molecular mechanisms of the clock have been subjects of thorough studies, especially in the last 20 years. A series of key studies ^2, 10– 12^ has shown that the core clock oscillator involves a fairly small set (likely in the order of 20) of genes, called core clock genes. These genes in turn regulate the temporal expression of other genes, which drive programs of 24 hour tissue-specific rhythms in gene expression and physiology ^13, 14^.

The circadian clock’s free running period is not exactly 24 hours. However, under normal physiological conditions where the organism is subject to external 24 hour periodic cues (Zeitgebers), the clock synchronizes to external time. An important consequence is that the internal positioning of an individual’s circadian timekeeper with respect to the external time varies from individual to individual, depending on the combination of free running period and amplitude of that individual’s clock and importantly the Zeitgeber strength, e.g. the intensity of light. The difference (dephasing) between the internal and external time is called chronotype and varies in humans with a standard deviation of 2 hours ^15– 19^.

## The importance of the clock for human health

A lot of attention has been drawn to the circadian clock in recent years as more links were found between various illnesses and a malfunctioning clock. In particular, during the last 20 years, two questions were repeatedly addressed: what are the most prominent effects on human health of a disrupted circadian clock, and what are the main causes in today’s lifestyle that deteriorate our internal timing system? Notably, the idea that the clock, due to reciprocal interactions with cell-cycle control, could be involved in a tumor suppressor function has existed since the end of the last century ^20^ and has been under scrutiny ever since. Faults in the circadian clock have also been connected with the aberrant metabolism of cancerous cells ^21^. However, it is only fairly recently that the role of the circadian clock in human health has earned a more prevalent position in biomedical research, as ties have been discovered with a wider variety of diseases. Studies have shown that in humans a perturbed circadian clock, due to shift work and sleep disruption, leads to metabolic pathologies ^22^. In mice, different aging-related phenotypes appear depending on which gear of the clock is broken; in particular, in some cases, lifespan is significantly shorter ^23^. In addition, the synchrony both among all the internal clocks in different organs and with the external cycle is responsible for proper timing of downstream metabolic processes, thereby contributing to the health of the organism; the interplay of the circadian and the metabolic networks can be disrupted, especially in humans, by a variety of factors such as aging, meal timing, jet-lag, and shift work ^24^. The issue of understanding exactly how light conditions and timing of food intake affect the daily metabolic and sleep cycles has been recently discussed in 25; this piece has also highlighted how circadian rhythms may be viewed as a new strategy to treat diseases in which anomalous metabolism is typical. With the modern abundance of available data on humans, it has been possible to establish that consistent alterations in the expression of circadian genes between healthy and diseased patients are prevalent ^26^. In addition, pharmacological modulations of the circadian clock have been introduced as a concept to fight cancer ^27^. In recent years, because of the vast availability of data, more and more connections between the circadian clock and cancerous growths have been identified ^28– 30^. To sum up, our current understanding suggests that the lack of a functioning circadian clock contributes to irregular and spread-out food intake and metabolic disorders and may lead to higher cancer rates and a shorter life span. However, it should be mentioned that, although promising in offering potential novel therapeutic avenues, complete biochemical proofs of these notions are still lacking in many cases. On the other hand, many factors contribute to the weakening of the circadian rhythm; some are due to our lifestyle, such as sleep disruption, shift work, and absence of 12 hour fasting periods, and others are further from our control, such as aging, chronic diseases characterized by altered metabolism, and cancer.

## Animal experiments around the clock, atlases, and benchmarks

To study the mammalian clock, many experiments spanning the full day have been performed, mostly on mice. The general setup is to sacrifice mice every 2 to 6 hours over 24 hours. Most of these experiments use highly controlled conditions. Notably, the mice are typically of one genetic background and taken from the same gender; moreover, environmental light or temperature conditions are tightly controlled, as well as the feeding regimen and schedules.

Although many experiments focus on specific organs or conditions, some transcriptome analyses provide comprehensive views on temporal gene regulation across an entire organism. Five years ago, the first atlas of gene expression around the clock was published ^31^. In this experiment, two mice were sacrificed every 2 hours, then 12 tissues were analyzed for their mRNA expression levels using RNA sequencing. This seminal dataset has since then served as a benchmark for other around-the-clock experiments on mice ^32^. This mouse dataset has also served as testing ground for a number of methods aiming to reconstruct circadian phase from single omics snapshot samples. Given the strictly controlled experimental conditions typical of mice experiments, this atlas minimizes all the variation in gene expression due to factors different from the circadian clock. More recently, an atlas on baboons ^33^ has been published in an effort to close the gap between mice and humans. In this case, one baboon was sacrificed every 2 hours and 64 tissues were collected and analyzed. As baboons are more similar to humans than mice, these data may in principle provide a better benchmark for future circadian studies in humans, especially considering the genetic diversity of the sacrificed baboons. In particular, mice are nocturnal animals and there are thus intrinsic differences in clock programs ^34^, though these are not yet fully characterized. We note that some of the animal studies are powered to overcome false discovery rate (FDR) corrections, while others are not.

## Computational methods to infer circadian phase

**Figure 1.**
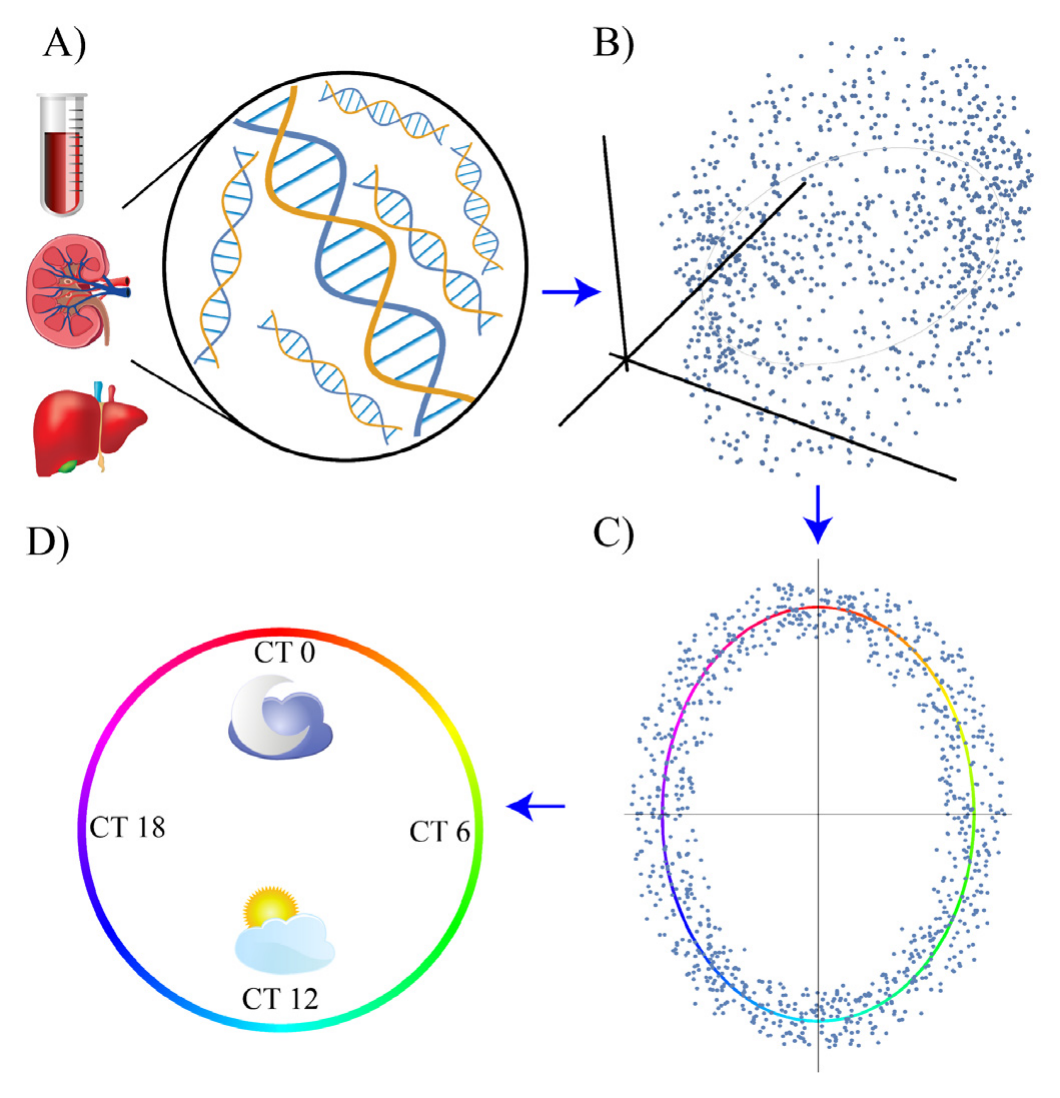
A summary of the steps followed by many phase reconstruction algorithms. The first step is the bulk mRNA extraction and sequencing from omics samples ( **A**); thus, high-dimensional data are generated ( **B**). The data are then projected in a low-dimensional representation and the circadian trajectory is identified ( **C**). Lastly, the data are projected onto the circadian trajectory and the internal time (circadian time, CT) of each sample is identified ( **D**).

**Figure 2.**
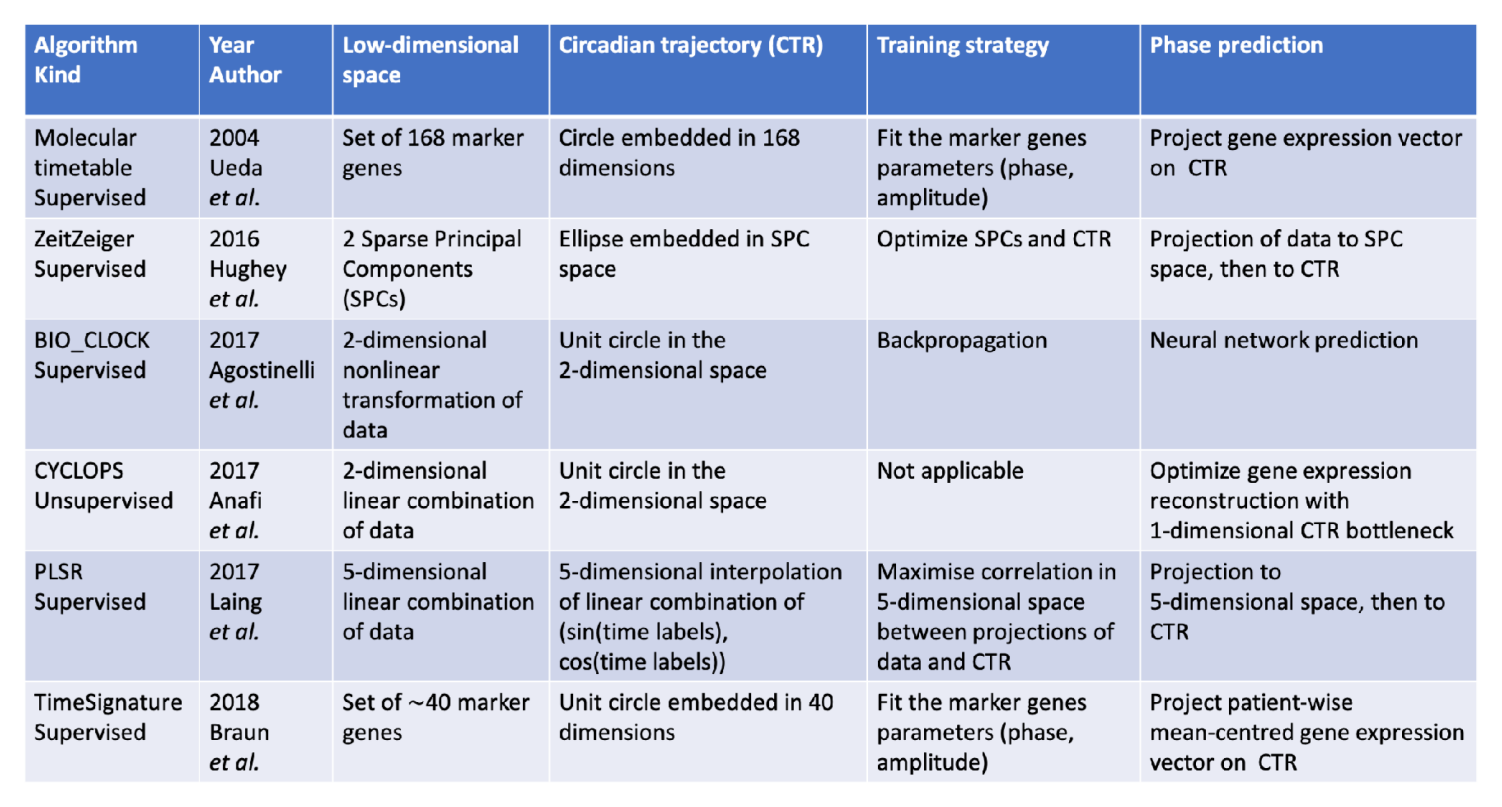
A summary of the most important characteristics of the existing methods to decode circadian phase from omics samples.

Since the importance of circadian timing in human health was established, an interesting question arose: how can we detect the internal time of a tissue sample from its gene expression level? Answering this question might open up significant novel medically relevant opportunities, especially for diagnosis, prognosis, and potentially therapeutic strategies ^35^ for a variety of illnesses, most importantly cancer. In addition, reliable methods to infer circadian phase would allow us to further study the structure and effects of the clock by leveraging the vast quantity of existing unlabeled RNA sequencing data on humans available in public databases, thus gaining important insights on clock biology in healthy and diseased human tissues, notably useful for future biomedical applications. Although many outstanding works have been recently published, how reliably and robustly this can be achieved remains an open question. An array of methods is currently available. The majority of algorithms available are supervised in the sense that the parameters are trained on time-labeled data (the training set). A word of caution concerns the intrinsic difference between the circadian phase and the external time, as explained above, which is generally not considered: circadian phase and the external time are often taken as equal in labeled datasets. One natural method to assign a circadian phase to unlabeled samples consists of applying the four-quadrant arc-tangent function to the two first principal components ^36^; we will refer to this unsupervised method as PCA. In 2004, a first significant step was made ^37^ by developing a method, molecular timetable, to infer the circadian phase based on the temporal and expression patterns of known clock-related marker genes in mice livers. The core idea is shared with several other supervised methods that will follow it. Namely, it consists of finding a suitable low-dimensional representation (also called the low-dimensional manifold) and then, in this coordinate system, finding the one-dimensional trajectory (termed here circadian trajectory) that best matches the known labels of the samples in the training set. A graphical representation of this core idea is shown in Figure 1. To find the phase of a new sample, it must be suitably projected onto the circadian trajectory in the low-dimensional space. In 37, the low-dimensional manifold consists of a set of standardized “time-telling” marker genes for which a sinusoidal shape is assumed and the peak phase fitted from the training set. The inferred circadian phase is obtained by minimizing the least square error across all the time-telling genes. Although simple, this method deserves praise for the pioneering work, also considering the scarcity of sequencing data available compared to modern day. For the next important contribution, we have to wait more than 10 years. The availability of data and the increased interest in the circadian clock attracted many researchers to this problem, which yielded four new methods, each claiming to be better than the last, in less than 4 years. The first was ZeitZeiger ^38^ in 2016. This method adds a supervised twist to PCA. The low-dimensional space is a set of sparse principal components (SPCs), each component being a sparse linear combination of genes. The linear combination of each component and the non-linear mapping from the SPCs to the circadian phase is optimized on the labeled training set, while the number of SPCs and the maximum nonzero entries in each of them are optimized by cross validation. To infer the phase of new samples, the algorithm linearly projects the gene expression levels to the SPC space and then finds the phase at which the circadian trajectory is closest to the projected gene expression levels. This method exhibits good performance in inferring the circadian phase; in addition, because of its easy and clear structure, it is able to identify which genes carry the most information about the circadian oscillation in a labeled sample. A few months after, a machine learning method was developed: BIO_CLOCK ^14^. This method is made of a three-layer dense neural network taking as input the normalized expression of 16 clock genes and yielding as an output the sine and cosine of the circadian phase. This method was trained and tested on mice microarray data from different organs with a 70–30 training-test split. Being a dense neural network, the training can be performed via backpropagation. To infer the phase of a new standardized sample, one simply inputs the required gene expression levels and takes the four-quadrant arc-tangent of the network’s output. Although this method uses a black box neural network, its performance is on a par with ZeitZeiger. Finally, the need for an unsupervised method (no labeled dataset is available) was recognized, and in 2017 CYCLOPS was invented ^39^. This method uses a linear autoencoder with a circular node, constructed with two coupled neurons, so the low-dimensional space is the two-dimensional space of the code neurons and the circadian trajectory is the unit circle in the two-dimensional space from which the circadian phase is extracted via the arc-tangent. CYCLOPS does not need a training set to learn its governing parameters; however, it cannot predict the phase of only one new sample. The general scheme of this algorithm is as follows: project to a low-dimensional space, from this project to a circadian trajectory, and from the circadian trajectory try to project back onto the original space, making the smallest possible error across all samples. For CYCLOPS, the low-dimensional representation is reached by linear projection onto two dimensions and the circadian trajectory is simply the unit circle where all points of the two-dimensional space are orthogonally projected; from the two-dimensional unit circle, points are linearly projected back to try to match the measured gene expression levels. The difference between CYCLOPS and PCA is the non-linearity from the two-dimensional space to the one-dimensional circadian trajectory included in the autoencoder. CYCLOPS offers one crucial advantage over supervised methods: it is more easily generalizable. This means that it can be directly applied to a much wider range of datasets, even ones with biologically different periodic behaviors, like the cell cycle, without the need for enough labeled data to retrain the algorithm. However, one must note that to obtain the best results the input of CYCLOPS should be restricted to a subset of genes, which have been previously implicated with time. Still, in 2017, a new supervised method, PLSR ^40^, was published. The idea of this method is to linearly project both the sequencing data and the two-dimensional representation of the phase (its sine and cosine) onto a five-dimensional feature space and maximize the correlation between the two. In this case, the projection to the feature space is optimized on the training set and the dimensionality of the feature space is optimized via cross validation. This method was built, trained, and tested on in-house blood sample measurements, which, unlike all the other training sets, used the dim light melatonin onset (DLMO), the gold standard assay to estimate circadian phase in humans, as the measure for circadian phase, not the external time. Using the gold standard for circadian phase detection in the training set gives this method a good advantage; however, it was never applied to datasets not collected in the authors’ lab. Lastly, another supervised method to infer circadian phase from blood RNA sequencing data was developed: TimeSignature ^41^. This approach is reminiscent of 37, as the low-dimensional space consists of a subset of genes, then, using the training set sinusoidal patterns are fitted for each gene with a ridge and lasso penalization to control the amplitude of each gene and reduce the number of genes kept. To infer a new phase, one must plug the gene expression level and determine the best fitted phase from the shapes of the reference genes. This method is trained and tested on human blood, with external time as the label. In addition, there is one disadvantage: each sample needs to be paired with another one, from the same person, taken 10–14 hours apart; without this pairing, the method cannot work, as the first step is a within-subject renormalization. This renders the method inapplicable to most existing data and deeply constrains its use in a medical context. The latest, still unpublished, method, TimeTeller, is a supervised method that exploits the periodic expression and covariance of 10–15 key genes to infer external time from a single tissue sample ^42^. A summary of the main characteristics of each of these methods is presented in Figure 2. Although it is generally required for methods of the same category (supervised, unsupervised) to show that they perform better than their predecessor, this fact needs to be taken with a pinch of salt. A common issue with comparisons between methods is that all methods are built on different datasets and optimize a slightly different quantity which further bias any comparison.

## Applications

Finally, we dedicate a few words to the impact that some of these methods had and how they have been applied. As we have seen, ZeitZeiger is a supervised method originally trained on various mouse tissues; in 2017, it was trained on existing whole human blood microarray data and a small set of marker genes was found to reliably reconstruct the circadian phase of each sample ^43^. A year later, in another study using blood samples, ZeitZeiger was trained on monocytes purified from the blood to accurately obtain the circadian phases from single blood samples ^44^. The resulting improved accuracy can be explained by the fact that peripheral blood mononuclear cells (PBMCs), which are used by most studies, consist of a complex mixture of many cells including T and B cells, which together make up more than 80% of the cells; however, the clock in T or B cells is weak. On the other hand, monocytes host a high amplitude clock, which likely explains the superior accuracy at predicting the DLMO. Other researchers focused on human skin: in a first step, they applied CYCLOPS to reorder a large set of unlabeled samples, then they applied ZeitZeiger to find a small subset of biomarkers for circadian phase in human skin ^45^. Application of these algorithms to data from blood or skin samples is particularly relevant, as they are easily accessible and can potentially be easily used in biomedical research. Of note, the different studies listed differ by the type of readouts used (candidate genes vs. whole genome); in the context of clinical applications, it appears that candidate gene approaches such as NanoString assays ^44^ might have a clear advantage in terms of costs and complexity. An interesting application of these methods was published in 2018. CYCLOPS was applied to temporally order human RNA sequencing data from 13 different post-mortem tissues and used to perform a comparative rhythmic analysis across them ^46^. In particular, the authors focused on drug target genes to propose improvements in the effectiveness of therapies by delivering drugs at the optimal time of day. In conclusion, the discussed advances in biomedical circadian biostatistics application promise to sprout important advances in around-the-clock treatments, especially those concerning the maximization of drug efficacy and the minimization of their side effects ^47^.
